# Determining muscle plasticity and meat quality development of low-input extended fed market-ready steers

**DOI:** 10.1093/tas/txae064

**Published:** 2024-05-02

**Authors:** Jordan C Wicks, Alexis L Wivell, Mariane Beline, Morgan D Zumbaugh, Jocelyn S Bodmer, Con-Ning Yen, Chantal Johnson-Schuster, Thomas B Wilson, Scott P Greiner, Sally E Johnson, Tim H Shi, Saulo Luz Silva, David E Gerrard

**Affiliations:** School of Animal Sciences, Virginia Polytechnic Institute and State University, Blacksburg, VA 24061, USA; School of Animal Sciences, Virginia Polytechnic Institute and State University, Blacksburg, VA 24061, USA; School of Animal Sciences, Virginia Polytechnic Institute and State University, Blacksburg, VA 24061, USA; Department of Animal Sciences and Industry, Kansas State University, Manhattan, KS 66506, USA; School of Animal Sciences, Virginia Polytechnic Institute and State University, Blacksburg, VA 24061, USA; School of Animal Sciences, Virginia Polytechnic Institute and State University, Blacksburg, VA 24061, USA; School of Animal Sciences, Virginia Polytechnic Institute and State University, Blacksburg, VA 24061, USA; School of Animal Sciences, Virginia Polytechnic Institute and State University, Blacksburg, VA 24061, USA; School of Animal Sciences, Virginia Polytechnic Institute and State University, Blacksburg, VA 24061, USA; School of Animal Sciences, Virginia Polytechnic Institute and State University, Blacksburg, VA 24061, USA; School of Animal Sciences, Virginia Polytechnic Institute and State University, Blacksburg, VA 24061, USA; Department of Animal Science and Food Engineering, College of Animal Science and Food Engineering, University of Sao Paulo, Pirassununga, SP, 13635-900, Brazil; School of Animal Sciences, Virginia Polytechnic Institute and State University, Blacksburg, VA 24061, USA

**Keywords:** Beef quality, color, forage, grain, market-ready, muscle plasticity

## Abstract

In March 2020, the World Health Organization declared COVID-19 a pandemic, which ultimately led to many meat processors temporarily shutting down or reducing processing capacity. This backlog in processing capacity forced many feedlots to retain cattle for longer periods of time and assume the risk of major market fluctuations. The aim of this study was to understand how a dietary insult affects meat quality and muscle metabolism in market-ready steers (590 kg). Sixteen market-ready (590 kg) commercial Angus crossbred steers were subjected to a maintenance diet of either forage or grain for 60 d. Longissimus lumborum (LL) muscle samples were collected immediately postmortem and processed for characteristics reflecting the underlying muscle fiber type and energy state of the tissue. Despite cattle being subjected to a 60-d feeding period, there were no detectable differences (*P* > 0.05) in carcass characteristics, color of lean, or ultimate pH (pH_u_). Moreover, our data show that muscle plasticity is rather resilient, as reflected by lack of significance (*P* > 0.05) in oxidative and glycolytic enzymes, myosin heavy chain isoforms (MyHC), myoglobin, and mitochondrial DNA (mtDNA) contents. These data show that market-ready steers are capable of withstanding a low-input feeding strategy up to 60 d without dramatically impacting underlying muscle characteristics and meat quality development.

## Teaser

Retained market-ready cattle can maintain carcass yield and quality up to 60 d when subjected to low-input maintenance diets of forage or grain.

## Introduction

Historically, consumers prioritized intrinsic cues, such as nutrition and safety when making decisions at the meat counter (Schroeder et al., 2013). Today, consumers make purchasing decisions with an added focus on socially conscious benefits, such as origin, animal welfare, and sustainability claims (Aboah and Lees, 2020). Despite the addition of these novel cues, meat quality is still important to consumers seeking a positive eating experience (Liu et al., 2022). Visual indicators such as meat color and degree of marbling are often leading factors for consumers when making purchasing decisions (Aboah and Lees, 2020). The U.S. Department of Agriculture (USDA) created Beef Grading Standards in 1927. This program was designed to evaluate beef carcasses based on age, lean color, and degree of marbling in an effort to segregate beef into more uniform classifications (USDA-AMS, 2017). Additionally, this system was an attempt to brand various levels of quality in effort to better advise consumers on predicted palatably, as well as create a unified pricing system for producers, processors, and consumers. Carcasses that achieve the grade of Prime or Choice are often a result of intensive feeding strategies, and while easily marketable to consumers, it requires greater inputs. Even so, the beef industry is a consumer-driven market. Therefore, cattle that are adequately fed and achieve optimal quality, or qualify for branded programs such as “All Natural” are rewarded with premiums (USDA-AMS, 2023), ultimately incentivizing producers to produce “fed” cattle. Conversely, producers are penalized for producing cattle that are older, possess lower quality, or yield dark lean. This system of purchasing beef carcasses is commonly referred to as “grid” pricing (Schroeder et al., 2009). Despite the financial incentives grid pricing provides, markets fluctuate, creating both “bull” and “bear” markets. However, during periods of bear market, producers are often faced with selling assets at inopportune market prices or continue to increase feeding cattle on expensive diets, waiting to sell during a more profitable market. Unfortunately, due to grid pricing, the latter option results in increased yield grade as well as heavier carcass weight, which can result in added discounts, only further exacerbating feedlot marketing decisions.

Although balancing feeding strategies and quality endpoints with the timing of the market is a constant reality for producers, the pandemic highlighted the challenges producers face when producing high-quality, profitable beef. Once the World Health Organization declared COVID-19 a pandemic (World Health Organization, 2020), much of the US went into immediate lockdown. The increased need for social distancing forced many meat processors to shut down or reduce processing capacity to stem plant outbreaks (Marchant-Forde and Boyle, 2020). Harvesting beef in the US from April to June 2020 was reduced by nearly 25% to 35% compared to the year prior (Lusk et al., 2021). This backlog in processing forced many feedlots to retain cattle for longer periods of time, increasing their days on feed (DOF; Halfman, 2020). In fact, feedlots with a capacity of 1,000 head or more reported an increase of 252% of cattle on feed for greater than 180 d (Schulz, 2020). This increase in retention required producers to alter normal production strategies in an effort to mitigate financial loss and slow growth rates. Even so, average carcass weights increased by nearly 25% (Martinez et al., 2021), with carcasses approaching 408 kg, qualifying for a discount in grid pricing (USDA-AMS, 2023). Granted, the degree of marbling in retained cattle also significantly increased (USDA-AMS, 2021). Still, with carcasses dressing over 408 kg, premiums for quality grade are often not sufficient to overcome discounts on Hot carcass weight (HCW) and yield grade due to excessive trim and oversized retail cuts (Reiman, 2020). This “holding” of finished cattle, though necessary at the time, ultimately cost the feedlot sector over $3 billion within the first month of the declared pandemic (Peele et al., 2020).

While the pandemic was unforeseeable and nearly impossible to circumvent, it exposed the US to serious vulnerabilities in our food supply. Furthermore, it forced producers across the industry to shoulder the financial burden of retained ownership and assume the risk of major market fluctuations. Although global pandemics are the exception rather than the rule, fluctuations in the markets still occur that cause operations to sell cattle at inopportune times. Hence, there is a need to better understand the resilience of market-ready steers (590 kg) so operations have more flexibility in marketing their assets. Therefore, the aim of this study was to investigate muscle’s response to dietary insult as it relates to beef quality and yield.

## Materials and Methods

All animal experimental procedures were approved by the Virginia Tech Institutional Animal Care and Use Committee under protocol number 18-180. Experimental animals were managed according to the guidelines outlined in the Guide for the Care and Use of Agricultural Animals in Research and Teaching (FASS, 2020).

### Animals and Treatments

Twenty-two Angus crossbred steers of similar body weight (BW) and age (BW = 416 ± 40 kg, age = 15 ± 2 mo) were fed a grain-based finishing diet (Table 1) for 108 d at the Virgina Tech Beef Center in Blacksburg, VA. Steers were grazed on tall fescue pastures and offered 1.86 kg/d of corn gluten feed pellets for 152 d between weaning and being placed on the finishing diet. The finishing diet contained 38.4% dried distillers grains plus solubles (DDGS), 38.3% cracked corn, 21.3% dairy total mixed ration (TMR) feed refusal, 1.2% custom mineral mix, and 0.8% ground limestone. Dairy TMR feed refusal was used as the forage source in the finishing diet because of limited haylage and silage supplies and was from the lactating cow TMR fed at the Virginia Tech Dairy Science Complex. The finishing diet was formulated using the NASEM (2016) model with a target metabolizable energy allowable gain of 1.58 kg/d. Cattle were offered ad libitum access to the finishing diet with dry matter intake (DMI) adjusted using the 4-point feed bunk scoring system described by Lundy et al. (2015). Average steer BW was 604 ± 53 at the conclusion of the finishing period.

**Table 1.** Common finishing, grain-based maintenance, and forage-based maintenance diets fed to finished market steers.

	Treatment diets^1^		
Item	Finishing	Forage maintenance	Grain maintenance
Ingredient, % DM			
DDGS	38.4	—	40.0
Cracked corn	38.3	—	39.0
Tall fescue/alfalfa haylage^2^	—	98.7	—
Dairy TMR feed refusal^3^	21.3	—	18.0
Custom mineral^4^	1.2	1.3	2.00
Ground limestone	0.8	—	1.00
Analyzed nutrient content			
DM, % as fed	71.8	67.9	67.9
CP, % DM	19.1	18.5	18.9
NDF, % DM	22.5	50.7	23.2
ADF, % DM	9.6	34.7	9.4
EE, % DM	5.8	2.8	5.1
Ash, % DM	5.9	10.7	6.8
NE_m_, Mcal/kg	2.1	1.38	2.1
NE_g_, Mcal/kg	1.4	0.82	1.4
Avg. DMI, kg/d	13.1	8.9	5.8

^1^Common finishing diet was fed to all cattle during 108 d finishing period that ended on day 0. Forage and grain maintenance diets fed diets were formulated to meet maintenance requirements and fed from days 0 to 60.

^2^Forage switched to alfalfa haylage on day 14 of maintenance period to reduce observed feed refusals associated with tall fescue haylage.

^3^Feed refusal from the lactating cow total mixed ration fed at the Virginia Tech Dairy Science Complex.

^4^Custom mineral (Southern States Cooperative, Richmond, VA) contained: 20.0% salt, 13.0% Ca (CaCO_3_ and CaHPO_4_), 2.0% P (CaHPO_4_), 12.0% Mg (MgO and MgSO_4_), 1.0% K (KCl and K_2_SO4), 0.5% S (K_2_SO4, MgSO_4_, FeH_2_O_5_S, and CoSO_4_), 1,300 mg/kg Zn (ZnO), 1,200 mg/kg Mn (MnO_2_), 300 mg/kg Cu (C_4_H_16_Cl_2_CuN_4_), 60 mg/kg I [Ca(IO_3_)_2_], 50 mg/kg Co (CoCO_3_ and CoSO_4_), 25 mg/kg Se (Na_2_SeO_3_), 220,462 IU/kg vitamin A, 55,115 IU/kg vitamin D, 551 IU/kg vitamin E, and 200 mg/steer·day^−1^ monensin (Rumensin 90, Elanco Animal Health).

At the end of the finishing period (day 0), 6 steers (603 ± 76 kg) were randomly selected and harvested to serve as a baseline for carcass and quality assessments, as well as color and ultimate pH (pH_u_) of typical market-ready cattle. Remaining steers (*n* = 16) were randomly assigned to maintenance diets with either forage (haylage; *n* = 8) or grain (*n* = 8) as primary energy sources for 60 d (Table 1). The grain-based maintenance diet contained 40% DDGS, 39% cracked corn, 18% dairy TMR feed refusal, 2% custom mineral mix, and 1% ground limestone. The forage-based maintenance diet contained 98.7% haylage and 1.3% custom mineral mix. Maintenance diets were limit-fed and formulated using the NASEM (2016) model with a target metabolizable energy balance of 0 Mcal/d so that energy supply was similar between treatments. Tall fescue was used in the forage maintenance diet from days 1 to 13 of the maintenance period. The forage source in the forage maintenance diet was switched to alfalfa haylage on day 14 to reduce observed feed refusals associated with the tall fescue haylage. The forage nutrient analysis presented in Table 1 reflects a weighted, composite sample of the tall fescue and alfalfa haylages.

During the finishing period, cattle were housed in two 0.40 ha drylot pens (11 steers per pen). Drylot pens were open, unpaved lots with a row of trees to provide a windbreak. Cattle were fed once daily in concrete, fence line bunks. Pen and feed bunk space exceeded FASS (2020) guidelines. On day 1 of the maintenance period, remaining steers were re-penned by their treatment assignment (8 steers per pen). Cattle had ad libitum access to automatic waterers. Average DMI reported in Table 1 is the average DMI of all cattle fed a particular diet.

### Animal Growth Performance Measures

Empty BW was recorded before feeding at the start of the finishing period as well as days 0, 30, and 60 of the maintenance period. Average daily gain (ADG) was calculated for the 108-d finishing and 60-d maintenance periods. The BW recorded on day 0 of the maintenance period was used as the final finishing BW and initial maintenance BW measurement. Ultrasound 12th rib fat thickness (FT) was measured on days 0 and 30 to assess differences in body composition during the maintenance period. Ultrasound images were taken with an Aloka 500SV (Hitachi Aloka Medical America, Inc., Wallingford, CT) B-110 mode instrument equipped with a UST-5011 3.5-MHz 125 mm linear array transducer. Measurements of 12th rib FT were taken in a transverse orientation between the 12th and 13th ribs approximately 10 cm distal from the midline. The distance function was used to measure the subcutaneous fat layer on frozen ultrasound images until three measurements within 0.07 cm were recorded.

### Harvest and Tissue Sample Collection

Following the 60-d feeding period, steers were randomly allotted to one of 4 d of harvesting (days 60, 61, 62, and 63) and transported to the Virginia Tech Meat Center for harvesting using standard protocols. Animals were allowed lairage and access to water before being harvested. Steers were weighed immediately before harvest to calculate dressing percentage (DP). Steers were rendered unconscious with a captive bolt and exsanguinated. Blood was collected during the exsanguination process, and muscle samples were collected from the longissimus lumborum (LL) muscle immediately following exsanguination. Samples were snap-frozen in liquid nitrogen and stored at −80 °C for further analyses (time 0). HCW was recorded and all carcasses entered a conventional chilling cooler at approximately 45 min postmortem. Following a 24-h chilling period, an additional LL muscle sample was taken. Regardless of time point, muscle samples were diced, snap-frozen in liquid nitrogen, and stored at −80 °C until further analysis.

### Carcass Evaluation and Color Analysis

Following a 24 h chilling period (2 ± 1°C) carcasses were ribbed between the 12th and 13th rib for carcass evaluation, as described by American Meat Science Association (AMSA) yield and quality grading standards (AMSA, 2001). Ribeye area (REA), 12th rib FT, estimated percent kidney, pelvic, and heart fat (KPH), and HCW were measured and used to calculate DP carcass yield grade. Carcass maturity and marbling scores were used to determine carcass quality grade. Ribbed carcasses were allowed to bloom for 30 min prior to objective color analysis. Triplicate color measurements were taken using a Konica Minolta CR-400 colorimeter (Ramsey, NJ, USA), Illuminant D, 0^o^ observer angle. Averaged color values were expressed as Commission Internationale de l’Éclairage (CIE) L* (lightness), and a* (redness) b* (yellowness).

### pH Analysis

Muscle pH was measured as outlined by Bendall, (1973) with modifications. Finely ground tissue was homogenized in a buffer (1:8 w/v) containing 5 mM Na-iodoacetic acid and 150 mM KOH using a Qiagen Tissuelyser for 2 min at 25 1/s frequency. Once homogenized, samples were heated at 25 °C for 5 min, centrifuged for 5 min at 13,000 × *g*, and placed back on the heating block at 25 °C for 1 min. The pH was measured using a calibrated Orion Ross Ultra pH electrode (Thermo Scientific, Pittsburgh, PA).

### Blood Collection and NEFA Analysis

Blood was collected using BD Vacutainer Plastic Serum tubes (Fisher Scientific Cat Number: 23-021-018). Blood samples were allowed to clot at ambient temperature for 1 h and then centrifuged at 4 °C for 30 min at 1,100 × *g*. Serum was separated into 1-mL aliquots and stored at −80 °C for subsequent analyses of non-esterified fatty acid (NEFA). NEFA concentration was determined using a commercial quantitative colorimetric assay kit (Wako Diagnostics, Richmond VA), measured using a 96-well microplate and read at 550 nm. Final NEFA concentration was equated using the following equation:

Sample Concentration = Standard Concentration × (Sample Absorbance) (Standard Absorbance), and reported as mmol/L.

### Protein Extraction and Determination

One hundred mg of frozen powdered tissue (LL, heart, masseter (MS), cutaneous trunci (CT), liver) was homogenized in 8 M urea, 2 M thiourea, 3% SDS (w/v), 75 mM DTT, 0.05 M Tris–HCl (pH 6.8), and heated at 95 °C (Warren et al., 2003). Homogenized samples were diluted 1:20 and used for total protein quantification using Reducing Agent and Detergent Compatible Protein Assay (Bio-Rad Laboratories, Hercules, CA, USA), according to manufacturer’s specifications. Samples were diluted in extraction buffer (Warren et al., 2003) containing 0.05% bromophenol blue to a final concentration of 3 mg/mL. All samples were stored at −80 °C until further analysis.

### Gel Electrophoresis and Immunoblotting

Muscle proteins and controls were separated by SDS-PAGE (10%, 15%, or 18%), transferred to nitrocellulose membranes, and blocked at room temperature with either Prometheus OneBlock Blocking buffer (Genesee Scientific Corporation, El Cajon, CA) or 5% nonfat dry milk in Tris-buffered saline solution with 0.1% tween-20 (1 × TBS-T) added. Following blocking, membranes were incubated overnight at room temperature with primary antibodies specific for phosphofructokinase-1 (PFK, Santa Cruz Biotechnology, Inc, SC-166722 at 1:1000 dilution), calpain-1 (CAPN1; Thermo-Fisher 9A4H8D3 at 1:1000 dilution), calpastatin (CAST; Thermo-Fisher 1F7ED10 at 1:1000 dilution), citrate synthase (CS; Santa Cruz Biotechnology, Inc, SC-390693 at 1:1000 dilution), succinate dehydrogenase-a (SDH-a; Abcam ab14715 at 1:1000 dilution), lactate dehydrogenase-A (LDH; Novus NBPI48336 at 1:30 000 dilution), O-linked β-N-acetylglucosamine (O-GlcNAc; Abcam ab2739 at 1:1000 dilution), and myoglobin (Santa Cruz Biotechnology, Inc, SC-25607 at 1:1000 dilution). IRDye fluorescent secondary antibodies (LI-COR Biosciences, Lincoln, NE) were used for visualization of bands, and protein abundances were normalized to total protein (Revert 700 Protein Stain, Li-Cor Inc., Lincoln, NE). All blots were imaged using a LI-COR Biosciences Odyssey Infrared scanner (Li-Cor, Inc., Lincoln, NE, USA) and band intensity was measured using Image Studio Lite (Li-Cor, Inc., Lincoln, NE, USA) with protein abundance reported as arbitrary units (AU).

### Gene Expression

Total RNA was extracted using the Direct-zol RNA Mini Prep Kit (Zymo Research, Irvine, CA). Twenty ng/µL of total RNA was reverse transcribed using the High-Capacity cDNA Reverse Transcriptase Kit (Applied Biosystems, Waltham, MA). About 2 µL of cDNA was used for amplifying gene-specific primers (Table 2) and SYBR chemistry in a 7500 Fast Real-Time PCR System (Applied Biosystems, Waltham, MA) for the quantification of myoglobin and myosin heavy chain (MyHC) isoforms. Relative gene expression was calculated by the 2^–ΔΔCt^ method.

**Table 2.** Primer sequence used in quantitative reverse transcription—PCR assays.

Gene Name	Sequence
*MHC I*	F5: AAA-GCT-AGC-CCA-GCT-GAT-TAC
	R5: CTC-TCT-CCT-CTC-CAC-CAT-CTT
*MHC IIA*	F1: TCT-GAA-CTC-TGC-TGA-CCT-ACT-C
	R1: CTG-CAT-TGG-TTA-CCT-GCT-CTA-C
*MHC IIX*	F5: AAA-GCT-AGC-CCA-GCT-GAT-TAC
	R5: CTC-TCT-CCT-CTC-CAC-CAT-CTT
*Myoglobin*	F1: CAG-GCT-CTT-CAC-AGG-TCA-TC
	R1: CCT-CAT-CTC-AGC-CTC-TGT-CTT-C
*S18*	F5: GCG-AGT-CAA-CAC-CAC-CAA-CAT-C
	R5: CCT-CAA-CAC-CAC-ATG-AGC-ATA-TC

### Mitochondrial DNA Content

Total DNA was purified using a DNAeasy mini spin columns according to manufacturer’s recommendation (Quigen, Germantown, MD) and quantified by optical density at 260 nm (Nanodrop 2000 spectrophotometer, ThermoScientific, USA). Mitochondria (mtDNA) and genomic DNA (gDNA) quantification were performed as previously described (López-Andreo et al., 2005). Briefly, 25 ng of total DNA was amplified (TaqMan Fast Advanced Master Mix Applied Biosystems) with organelle-specific DNA primers (500 nm each) and 250 nM MGB probe for 40 cycles of 20 s at 95 °C and 30 s at 60 °C for 40 cycles. Total mtDNA quantity (ng/μL) was inferred from the standard curve and normalized to the gDNA total quantity and presented as a ratio of the two as the fold difference.

### Feed Sampling and Analysis

Individual ingredient samples were collected every 9 to 20 d to update daily as fed feed intake and for subsequent nutrient analysis. Subsamples of each ingredient were dried at 105 °C for 24 h in a forced air oven to determine dry matter. The remaining sample was dried at 55 °C in a forced air oven for at least 72 h, ground to pass through a 1 mm screen using a Thomas Wiley mill (Thomas Scientific, Swedesboro, NJ). Ground samples of each ingredient for the common finishing and maintenance periods were composited for subsequent nutrient analysis.

Composite samples of fescue haylage, alfalfa haylage, and dairy TMR refusal were analyzed for crude protein (CP), neutral detergent fiber (NDF), acid detergent fiber (ADF), ether extract (EE), Ca, P, net energy of maintenance (NE_m_), and net energy of gain (NE_g_) by Cumberland Valley Analytical Services (Waynesboro, PA). CP, ADF, EE, and minerals were analyzed using AOAC (2000) methods 990.03, 973.18, 954.02, and 985.01 respectively. The method described by Van Soest et al. (1991) was used for analysis of NDF. Net energy values were calculated using the OARDC Summative Energy Equation that analyzed CP, NDF, ash, lignin, and EE values.

Composite corn samples were analyzed in the Virginia Tech Beef Nutrition Laboratory. Duplicate subsamples were analyzed for CP by measuring the percent nitrogen concentration using an Elementar Vario EL Cube (Elementar Americas Inc., Ronkonkoma, NY) and multiplying by 6.25 (AAOC method 990.03). Duplicate subsamples were analyzed for NDF and ADF using an Ankom 200 fiber analyzer (ANKOM Technology, Macedon, NY) following the manufacturer’s recommendations (AAOC method 973.18). The concentration of NDF was measured using the method described by Van Soest et al. (1991). Duplicate subsamples were analyzed for EE using an Ankom XT10 Extractor (ANKOM Technology, Macedon, NY) following the manufacturer’s recommendations (AAOC method 920.39). Ash was determined in duplicate by placing a 0.5-g sample in a furnace at 600 °C for 24 h. Net energy values were calculated from analyzed ADF concentrations. Analyzed ADF values were used to calculate percent Total Digestible Nutrients (TDN) using the corn-specific equations obtained from Rock River Laboratory (Watertown, WI) via personal communication: Net Energy of Lactation (NE_l_) = 0.9265 − (0.00793 ADF), TDN = 4.898 + (NE_l_ 89.796). Equations in the Energy Terms and Concepts chapter of NASEM (2016) were then used to calculate NE_m_ and NE_g_ from TDN. Composite DDGS were analyzed for NDF, EE, and ash using the same methods as for corn. Book values from NASEM (2016) were used for CP, ADF, NE_m,_ and NE_g._

### Statistical Analysis

Data were analyzed using the Proc Mixed procedure using SAS version 9.3 (SAS Institute Inc., Cary, NC, USA). Carcass was considered the experimental unit and the statistical model included the fixed effects of treatment. Harvest day was included as a random variable for meat carcass yield and quality variables. Means were compared using Tukey–Kramer Multiple Comparison Test if a significant treatment effect was detected. Data in results tables and figures are presented as least square means ± standard error means (SEM), and differences were considered significant at *P* < 0.05, or unless otherwise stated.

## Results

### Animal Growth Performance and Carcass Evaluation

To investigate the impact of low-input extended feeding on muscle plasticity and ultimate meat quality, independent of muscle growth and/or age, a maintenance diet of either forage or grain was fed to market-ready (604 kg) steers for 60 d. Measures of steer performance during the finishing and maintenance periods are shown in Table 3. There were no differences (P ≥ 0.465) in initial finishing BW, final finishing BW, or finishing ADG between cattle harvested as controls or those subsequently maintained on forage or grain-based maintenance diets. There were no treatment differences in BW (*P* ≥ 0.642) or ADG (*P* = 0.248) during the maintenance period. No observed differences (*P* ≥ 0.229) in ultrasound 12th rib FT on day 0 or day 30 of the maintenance period also indicate that body composition remained similar independent of BW.

**Table 3.** Least square means, standard error mean (±) and probabilities (*P* value) of the effect of treatment on live animal performance.

	Treatments^1^			
Item	Control	Forage	Grain	*P* value
BW, kg				
Start of finishing period	414.19 ± 17.26	409.59 ± 14.94	422.86 ± 14.94	0.819
Day 0 of maintenance period ^2^	603.28 ± 23.26	603.28 ± 20.14	604.70 ± 20.14	0.998
Day 30 of maintenance period	—	587.40 ± 14.50	580.88 ± 14.50	0.755
Day 60 of maintenance period	—	594.77 ± 16.47	605.83 ± 16.47	0.642
ADG, kg/d				
Finishing	1.75 ± 0.07	1.79 ± 0.06	1.68 ± 0.06	0.465
Maintenance	—	-0.14 ± 0.09	0.02 ± 0.09	0.248
Ultrasound 12th rib fat thickness, cm				
Day 0 of maintenance period	0.91 ± 0.10	0.91 ± 0.08	0.93 ± 0.08	0.986
Day 30 of maintenance period	—	0.85 ± 0.05	0.95 ± 0.05	0.229

^1^Control cattle finished on a grain-based diet for 108 d and slaughtered around day 0. Forage and Grain cattle-fed diets of differing energy sources formulated to meet maintenance requirements for and additional 60 d.

^2^BW recorded on day 0 of maintenance period used as final finishing BW when calculating finishing ADG.

Additionally, HCW (*P* = 0.507), DP (0.553), REA (*P* = 0.339), 12th rib FT (*P* = 0.800), KPH (*P* = 0.019) yield grade (*P* = 0.757), and marbling score (*P* = 0.133) was evaluated, with no differences noted between treatments with the exception of estimated percent KPH (Table 4).

**Table 4.** Least square means, standard error mean (±) and probabilities (*P* value) of the effect of treatment on carcass traits.

	Treatments ^1^			
Item	Control	Forage	Grain	*P* value
Final body weight, kg ^1^	586.64 ± 29.10	590.35 ± 29.10	613.11 ± 29.10	0.763
Hot carcass weight, kg	333.07 ± 15.35	340.31 ± 13.24	356.75 ± 13.29	0.507
Dressing percentage, %	56.78 ± 1.63	57.95 ± 1.63	59.34 ± 1.63	0.553
Ribeye area, cm^2^	88.26 ± 3.21	82.13 ± 2.77	84.39 ± 2.77	0.399
12th rib fat thickness, cm	1.33 ± 0.24	1.31 ± 2.77	1.27 ± 0.21	0.800
Estimated percentage KPH, %	1.41^ab^ ± 0.11	1.38^b^ ± 0.09	2.01^a^ ± 0.09	0.019
Yield grade	2.57 ± 0.30	2.83 ± 0.26	2.88 ± 0.26	0.757
Marbling score^2^	446.67 ± 29.56	477.50 ± 25.60	527.50 ± 25.60	0.133

^1^Control cattle finished on a grain-based diet for 108 d and slaughtered around day 0. Forage and Grain cattle-fed diets of differing energy sources formulated to meet maintenance requirements for and additional 60 d.

^2^Shrunk BW recorded on morning of harvest after lairage. Used to calculated DP.

^3^Marbling score: 200 = traces, 300 = slight, 400 = small, 500 = modest, 600 = moderate.

## Color and pH

**Figure 1:**
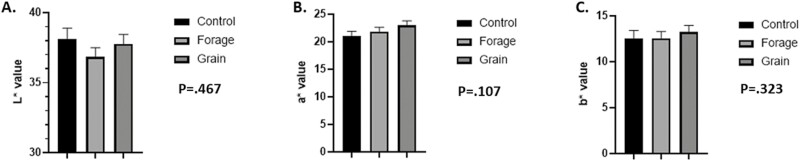
Lean color at 24 h. (A) Means of lightness (L*), (B) redness (a*), and (C) yellowness (b*) values LL at 24 h with 30 min bloom. Means are considered significantly different at *P* < 0.05.

**Figure 2:**
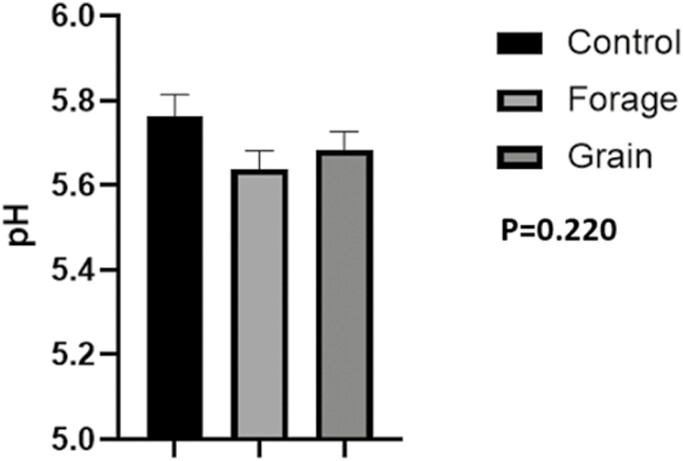
24 h pH of LL. Means of pH values from the LL at 24 h postmortem. Data represent LS means ± SE. Means are considered significantly different at *P* < 0.05.

No differences were noted in lightness (L*; Figure 1A, *P* = 0.467), redness (a*, Figure 1B, *P* = 0.107) or yellowness values (b*) (Figure 1C, *P* = 0.323). Moreover, there was no difference in pH at 24 h (Figure 2; *P* = 0.220) between treatments.

## NEFA Concentration and Protein Abundance

**Figure 3:**
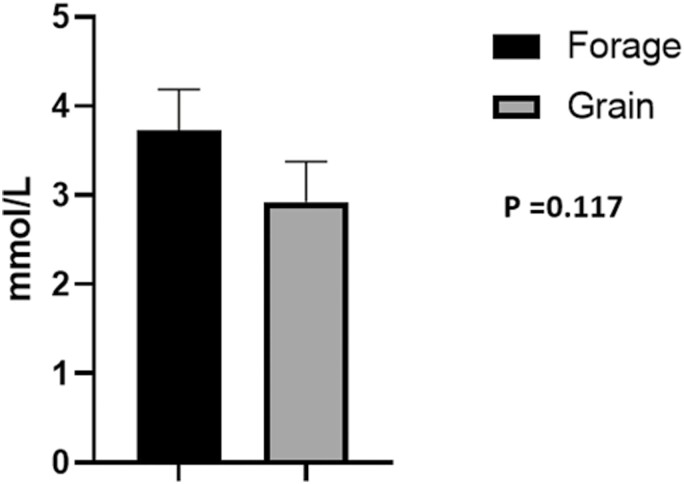
Non-esterified fatty acids. Means of non-esterified fatty acids (NEFA) concentration (mmol/L) between treatments post 60 d feeding trial. Data represent LS means ± SE. Means are considered significantly different at *P* < 0.05.

**Figure 4:**
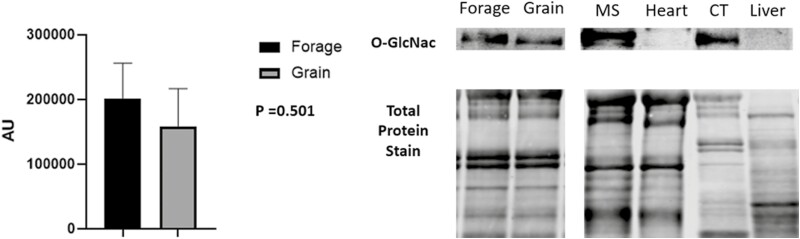
O-GlcNac abundance. Relative abundance of O-GlcNAc in longissimus muscle (LL) of forage and grain-fed cattle post 60 d feeding trial. Data represent LS means ± SE. Means are considered significantly different at *P* < 0.05.

**Figure 5:**
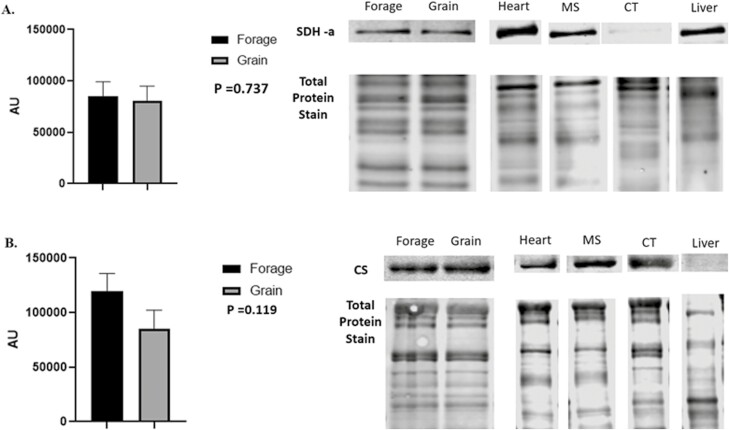
Abundance of oxidative biomarkers (SDH-A, CS). (A) Relative abundance of succinate dehydrogenase-A (SDH-A), and (B) citrate synthase (CS) in longissimus muscle (LL) of forage and grain-fed cattle post 60 d feeding trial. Data represent LS means ± SE. Means are considered significantly different at *P* < 0.05.

**Figure 6:**
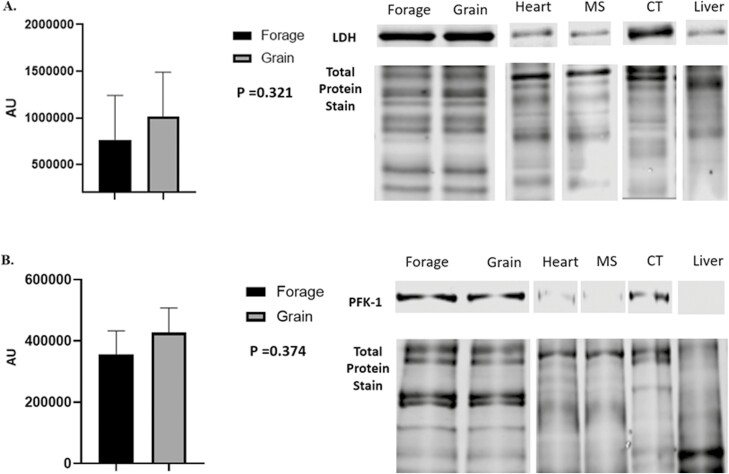
Abundance of Glycolytic Biomarkers (LDH, PFK). (A) Relative abundance of lactate dehydrogenase (LDH), and (B) phosphofructokinase-1 (PFK-1) in longissimus muscle (LL) of forage and grain-fed cattle post 60 d feeding trial. Data represent LS means ± SE. Means are considered significantly different at *P* < 0.05.

**Figure 7:**
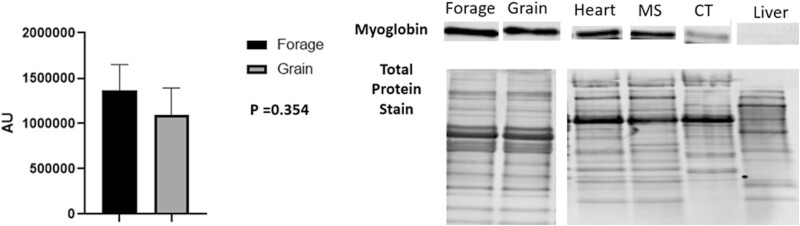
Abundance of myoglobin. Relative abundance of myoglobin in longissimus muscle (LL) of forage and grain-fed cattle post 60 d feeding trial. Data represent LS means ± SE. Means are considered significantly different at *P* < 0.05.

**Figure 8:**
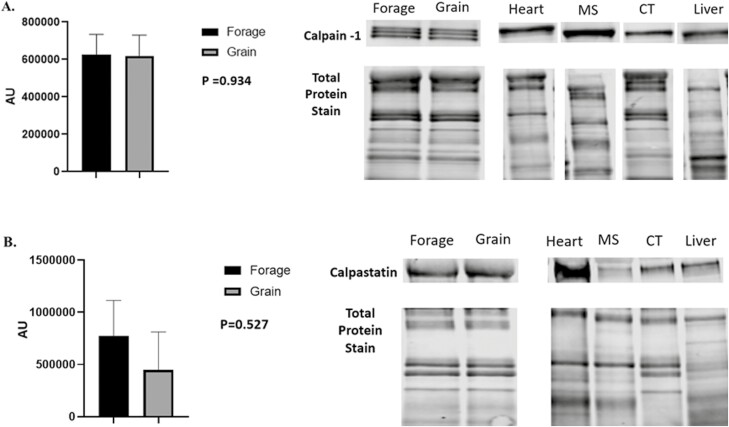
Abundance of Calpain-1 and Calpastatin. (A) Relative abundance of calpain-1 and (B) calpastatin in longissimu*s* muscle (LL) of forage and grain-fed cattle post 60 d feeding trial. Data represent LS means ± SE. Means are considered significantly different at *P* < 0.05.

Despite being subjected to differences in energy source for 60 d, no significant differences in NEFA concentrations were noted (Figure 3; *P* = 0.117). Underlying muscle characteristics were analyzed using western blotting assays. Overall, protein abundance tended to follow an expected outcome but no significant differences were noted in O-GlcNAc (Figure 4, *P* = 0.501), SDH-a (Figure 5A; *P* = 0.737), CS (Figure 5B; *P* = 0.119), LDH (Figure 6A; *P* = 0.321), PFK-1 (Figure 6B; *P* = 0.374), myoglobin (Figure 7; *P* = 0.354), calpain-1 (Figure 8A; *P* = 0.934), or CAST (Figure 8B; *P* = 0.527).

## Gene Expression and mtDNA

**Figure 9:**
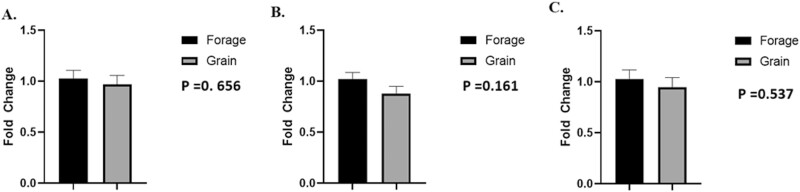
Gene expression of MHC-1, MHC-IIA, and MHC-IIX. Means of gene expression of (A) myosin heavy chain type I (MyHC-I), (B) myosin heavy chain type IIA (MyHC-IIA), and (C) myosin heavy chain type IIX (MyHC-IIX) between treatments and presented as fold differences. Data represent LS means ± SE. Means are considered significantly different at *P* < 0.05.

**Figure 10:**
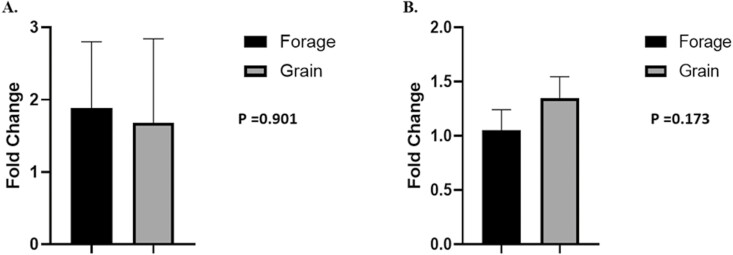
Gene expression of mtDNA, and myoglobin. (A) Mitochondrial (mt) DNA copy number relative to genomic (g) DNA copy number and (B) myoglobin gene expression between treatments and presented as fold differences. Data represent LS means ± SE. Means are considered significantly different at *P* < 0.05.

To understand the role of nutrient availability on muscle plasticity, gene expression of MyHC isoforms were evaluated. Furthermore, mtDNA abundance and myoglobin gene expression were compared between treatments in an effort to determine whether maintenance diet type changed muscle fiber type, as reflected by its oxidative markers. No differences were observed between treatments for MyHC-I (Figure 9A; *P* = 0.656), MyHC-IIA (Figure 9B; *P* = 0.161), MyHC-IIX (Figure 9C; *P* = 0.537), mtDNA abundance (Figure 10A; *P* = 0.901), or myoglobin (Figure 10B, *P* = 0.173).

## Discussion

Beef quality is a complex result of a number of different biological and biochemical processes that independently or together can affect the final product. At the same time, however, beef quality remains relatively stable as data reported herein suggest. Management strategies aimed at genetic selection, feeding regimes, and growth rate account for a number of differences in factors known for dictating ultimate quality. Specifically, breed, sex, plane of nutrition, and age can impact finishing endpoint, which is largely influenced by muscle type, energy metabolism, and fat deposition (Wicks et al., 2019). As muscle grows it shifts from oxidative to glycolytic, or more simply, from red to white (Picard and Gagaoua, 2020). This shift in fiber type alters the mechanisms in which energy is “handled” in the cell for function. Red, oxidative fibers rely on aerobic metabolism for energy production (ATP), while white fibers generate ATP through anerobic glycolysis. This discrepancy in energy metabolism heavily influences both the rate and extent of pH decline postmortem. Oxidative muscles have shown to have equivalently similar glycolytic potential to that of glycolytic muscles (Kirkpatrick et al., 2023), still muscle pH_u_ remains elevated (England et al., 2016), partially explaining the noted color differences between red and white muscles, and even between species. Moreover, proteolysis is highly correlated with both muscle fiber type (Totland et al., 1988; Bhat et al., 2018) as well as pH decline (Hwang and Thompson 2001; Melody et al., 2004; Hopkins and Geesink, 2009; Lonergan et al., 2010; Bhat et al., 2018), lending some further understanding to the disparity in tenderness between muscle phenotype (Ramos et al., 2020). Although pH decline does not directly influence intramuscular fat deposition, marbling favors a glycolytic phenotype (Hocquette et al., 2012).

Currently, the industry has determined that cattle-fed high-energy cereal grain diets produce the most consumer-acceptable product as it allows for an increase in type II fibers, efficient growth rate, and acceptable fat accumulation. However, muscle plasticity is responsive to both nutrient availability and restriction. Early weaning (Scheffler et al., 2014), compensatory gain (Sainz et al., 1995) and back-feeding cull cows (Matulis et al., 1987; Pritchard and Berg, 1993) are prime examples of how nutrient energy sources can alter muscle, postmortem metabolism, and ultimate quality. Even so, much of the current literature surrounding feeding regimes and meat quality center around growing muscle, and are confounded by DOF, growth rate, or finishing weight (Muir et al., 1998; Apaoblza et al., 2020; Gómez et al., 2022). Although we do not argue the influence plane of nutrition has on muscle metabolism and meat quality, Yambayamba and Price (1991), found no difference in fiber type between cattle subjected to ad libitum feeding compared to cattle subjected to 2 mo of restricted feeding. On the other hand, cattle subjected to 4 mo of restricted feeding possessed a greater abundance of oxidative fibers, compared to other treatments. These data argue a “window of time” may exist where cattle can be subjected to restricted or low-energy diets without shifting from glycolytic to oxidative muscle fiber type or losing yield and quality standards. Therefore, we chose to investigate the effects of protracted feeding of either forage or grain-based maintenance diets to market-ready steers (590 kg) for 60 d in an effort to better understand the effects of low-input protracted feedings on meat quality attributes and the cellular mechanism controlling them.

Both nutrient restriction and over-feeding cause differences in BW, growth rate, energy metabolism, and ultimate quality (Sami et al., 2004), as do differences in nutrient energy source (Gómez et al., 2022). However, variability in carcass and quality dissipates when BW and growth rate are held constant regardless of plane of nutrition (Gómez et al., 2022). Our data confirms the latter construct, as we found no difference in BW (Table 3), or carcass yield and quality (Table 4) between low-input extended feeding regimes and suggests growth may be required for shifts in muscle metabolism, and ultimate quality development.

Moreover, plane of nutrition has proven to influence meat color. However, we were unable to detect significant differences in L* values between treatments (Figure 1A). These data are in conflict with those of Shibata et al. (2009), Vestergaard et al. (2000b), Apaoblaza et al. (2020), Bidner et al. (1981), and Gómez et al. (2022), all of which found forage-based feeding regimes to produce darker lean. Even so, those studies differ from the present study because we allowed for maximal growth to finishing (590 kg), rather than limited growth to final weight. Moreover, those studies had differing endpoints in BW, FT, marbling, age, or even combinations of these variables. Such discrepancies can alter the intrinsic biology of muscle, subsequently influencing ultimate meat color (Hughes et al., 2020; Ramanathan et al., 2020). Gómez et al. (2022) found darker lean in beef finished on pasture-based feeding regimes finished to similar weights, however, the authors acknowledged color inconsistencies between grass and grain-fed beef may have be exacerbated by growth rate and also be influenced by marbling (Hughes et al., 2020). Moreover, Apaoblaza et al. (2020) noted darker lean in grass-fed beef compared to conventional intensive feeding regimes as well. However, these steers were fed for 127 d, allowing for increased growth, marbling, and shifting of muscle metabolism with grain-fed steers possessing a more glycolytic phenotype, suggesting discrepancy in color may be a result of difference in muscle metabolism. However, in a comparably short feeding trial (85 d), similar to the present study, French et al. (2000) found no difference in L* when BW and FT were equal at time of slaughter, Additionally, Daly et al. (1999), reported no difference in lean color of restricted grain-fed steers compared to similar growth rate of grass-fed steers, suggesting that an interaction of growth and plane of nutrition must occur in order to create difference in lean color of grass and grain-fed beef.

Still, L* is highly correlated to pH with higher pH resulting in darker lean (Qiao et al., 2001). Moreover, L* tends to shoulder that of b* values, which is also highly correlated with pH decline (Meadus and MacInnis, 2000). Abril et al., (2001) found b* values, yellowness, to be a useful indicator for discriminating between the two pH groups, correctly segregating samples 86–95% of the time. However, we found no difference in pH (Figure 2) or b* values (Figure 1C) suggesting muscle metabolism was not influenced by differing planes of nutrition at 60 d.

Even so, grass-fed beef can still appear dark in color despite having no difference in pH_u_ compared to grain-fed beef (Moloney et al., 2022). Yet, even when difference in pH_u_ is noted and lean of grass-fed beef is darker; pH_u_ is still well within what is considered optimal and normal pH (Briskey,1964), and suggests other factors aid in color discrepancy between grass and grain-fed beef. Apaoblaza et al. (2020), reported increased myoglobin concentrations and darker lean in beef from grass-fed cattle compared to that of grain-fed cattle. Myoglobin is highly correlated to redness (a*), however, the authors also reported that grass-fed beef was less red. Interestingly, we found no difference in myoglobin abundance (Figure 7; 10b), or a* values between treatments (Figure 1B). While myoglobin abundance is imperative for increased pigment, the redox state is equally as vital. As oxygen penetrates the muscle it binds to deoxymyoglobin (DMb) converting it to oxymyoglobin (OMb) shifting the pigment from purple to red. To that end, redness values (a*) are positively correlated with myoglobin reductase activity (MRA) and negatively associated with relative content of metmyoglobin (MMb%; Wang et al., 2021). This well-regarded construct is often a result in difference in mitochondria abundance (fiber type) or more simply, muscle metabolism (Vestergaard et al., 2000a; Apaoblaza et al., 2020). While we found no difference in a* values in the present study, we also found no difference in expression of various muscle fiber type-specific contractile proteins (MyHC-I, MyHC-IIA, MyHC-IIX; Figure 9A–C) or mtDNA compared to genomic DNA (Figure 10A) and may explain varying results compared to current literature.

Muscle plasticity refers to a muscle’s ability to alter both structural and functional properties, in response to extrinsic factors such as growth or plane of nutrition. In fact, as muscle grows it experiences hypertrophy and results in muscle shifting from red to white. This shift in fiber type also requires a shift in the way a muscle produces energy, moving from oxidative metabolism to a glycolytic metabolism (Picard and Gagaoua, 2020). Even so, metabolism must have an adequate nutrient source to sustain such energy requirements. For example, oxidative metabolism is largely a function of the mitochondria (TCA cycle), requiring acetate in the form of acetyl-CoA to begin the process. On the other hand, energy can also be produced through anerobic glycolysis, which through a cascade of reactions, converts glycogen to lactate. Cattle receive 70% of their nutrient energy from volatile fatty acids (VFAs) in the form of acetate, propionate, or butyrate (Bergman, 1990). The ratio of VFAs in the rumen is largely dictated by plane of nutrition and is well documented that pasture or grass-fed cattle have a higher proportion of ruminal acetate, compared to grain-fed cattle which have an increased abundance of propionate and butyrate (Zhang et al., 2017). This difference in nutrient availability can stimulate a shift in fiber type, with extensively fed cattle producing more type I fibers, while intensively fed cattle produced more type IIA, and IIB fibers (Vestergaard et al., 2000b). This increase in oxidative fiber type also results in decreased glycogen and increased citrate synthase activity and darker lean (Vestergaard et al., 2000b). Granted, the study by Vestergaard et al. (2000b) allowed for increased weight gain, and possible differences in voluntary exercise, which have also been linked to altering muscle metabolism and generating darker lean (Priolo et al., 2002). Even so, there is strong evidence to support the notion that plane of nutrition influences ultimate lean quality, at least when metabolism is shifted between treatments (Apaoblaza et al., 2020). Therefore, we explored nutrient sensing indictors as well as proteins abundance of oxidative and glycolic enzymes.

Although VFAs were not measured in this study, there is substantial evidence to support that acetate is increased in cattle subjected to forage-based diets (Zhang et al. 2017) and lends to an increase of energy production through the citric acid cycle. However, cattle with oxidative metabolism, fueled by acetate, tend to be nutrient stressed and express higher NEFA concentration, when compared to high-energy grain-fed cattle (Blanco et al., 2011). In fact, increased NEFA concentration is often an indicator of increased beta oxidation, which functions to convert fatty acids into acetyl-CoA, allowing for increased energy production by way of the Krebs cycle. Therefore, we measured serum NEFA concentration in an effort to determine if differences in feeding regime resulted in differences in energy availability. While NEFA concentrations for forage-fed cattle were numerically increased, we found no significant difference between treatments (Figure 3). This is in agreement with Blanco et al. (2011), who found significantly increased NEFA concentration in young bulls subjected to pasture grazing compared to feedlot finishing. However, this significance was lost over duration of time on feed. Even so, our diets were formulated to meet maintenance energy requirements and may aid in explaining why significance was not observed in the current experiment.

Although we found no difference in serum NEFA concentration, steers were still subjected to two differing rations for 60 d, potentially leading to variance in acetate to propionate ratio between treatments and influencing substrate availability. Apaoblaza et al. (2020) reported an increase in the nutrient sensor O-GlcNAc in grain-fed cattle. While still not fully understood, it has been reported that O-GlcNAc is highly correlated to substrate availability (Lazarus et al., 2012). To that end, Apaoblaza et al. (2020) also determined that grass and grain-fed cattle differ energetically, further supporting muscle’s ability to sense and respond to nutrient availability. Still, cattle from this study were fed for twice as long as the cattle in the present study and suggests that longer feeding periods may be required, as we found no difference in the O-GlcNAc between treatments (Figure 4).

Additionally, we analyzed both oxidative and glycolytic proteins in effort to better understand if we were able to shift metabolism through differing nutrient energy source. Despite a 60 d feeding period, we were unable to detect any difference in oxidative proteins such as SDH-a, CS or the pigment protein myoglobin (Figure 5A and B; 7, respectively). Moreover, we also found no difference in glycolytic metabolism surrogate proteins, LDH or PFK-1 (Figure 6A and B) between forage and grain-fed diets. This of course, contradicts others who have reported differences in muscle fiber type and/or metabolism between intensively and extensively feed cattle (Vestergaard et al., 2000a; Apaoblaza et al., 2020). Furthermore, calpain-1 and CAST were evaluated as indicators of proteolysis (Ouali, 1990), but also predicators of tenderness, yet we found no difference between either protein (Figure 8A and B). Protein similarity suggests that even with aging, proteolysis would not differ, resulting in comparable eating experiences, at least in regard to tenderness.

## Conclusion

In conclusion, our data trend as expected and strengthens the argument that nutrient energy sources can influence muscle metabolism and ultimate quality development. However, our data also show that beef muscle is somewhat resilient to short-term nutrient insults, and the use of forage or limited grain feeding for 60 d to maintain animals prior to slaughter did not influence carcass parameters or meat quality, suggesting the creations of nutrient-induced differences in quality and yield require more aggressive feeding challenges. To that end, our data indicates that low-input feeding strategies, if strategically managed, can be used to leverage marketing of fed cattle for financial gains.
